# A Divergence-Free Wigner Transform of the Boltzmann Operator Based on an Effective Frequency Theory

**DOI:** 10.1021/acs.jpca.1c05860

**Published:** 2021-10-12

**Authors:** Jens Aage Poulsen, Gunnar Nyman

**Affiliations:** Department of Chemistry and Molecular Biology, University of Gothenburg, SE 405 30 Gothenburg, Sweden

## Abstract

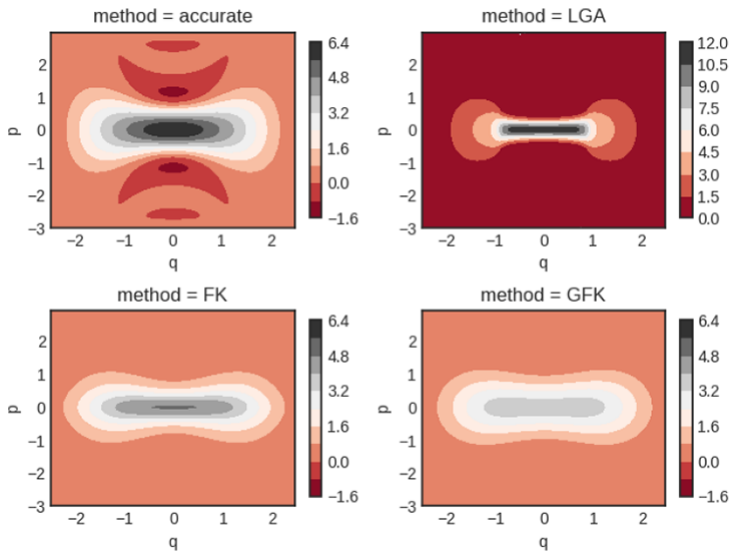

The centroid effective frequency representation of path integrals as developed by Feynman and Kleinert was originally aimed at calculating partition functions and related quantities in the canonical ensemble. In its path integral formulation, only *closed* paths were relevant. This formulation has been used by the present authors in order to calculate the many-body Wigner function of the Boltzmann operator, which includes also open paths. This usage of the theory outside of the original intention can lead to mathematical divergence issues for potentials with barriers, particularly at low temperature. In the present paper, we modify the effective frequency theory of Feynman and Kleinert by also including open paths in its variational equations. In this way, a divergence-free approximation to the Boltzmann operator matrix elements is derived. This generalized version of Feynman and Kleinert’s formulation is thus more robust and can be applied to all types of barriers at all temperatures. This new version is used to calculate the Wigner functions of the Boltzmann operator for a quartic oscillator and for a double well potential and both static and dynamic properties are studied at several temperatures. The new theory is found to be essentially as precise as the original one. Its advantage is that it will always deliver a well-defined, even if approximate, Wigner function, which can, for instance, be used for sampling initial conditions for molecular dynamics simulations. As will be discussed, the theory can be systematically improved by including higher-order Fourier modes into the nonquadratic part of the trial action.

## Introduction

The Wigner transform of the Boltzmann operator, introduced by Wigner almost a century ago,^1^ is an important tool for analyzing quantum effects in statistical mechanics; see, e.g., selected papers in ref (2). It has, for instance, served as an inexpensive tool for sampling quantized initial conditions for molecular dynamics simulations; see, e.g., refs (3−6).

Unfortunately, it is only for systems of very limited dimensionality that it is possible to accurately obtain the Wigner transform of the Boltzmann operator. Due to its usefulness, the Wigner transform and the problem of its computation is, however, the subject of intense ongoing research.^6−11^ Consequently, many approximate schemes for computing the Boltzmann Wigner transform also of large systems have now been put forth.^3,5,6,12−16^ Many of these methods rely on introducing various harmonic approximations in the otherwise intractable mathematical expressions.^3−6,12^ This enables a sufficient simplification of the equations so that an approximate, multidimensional Wigner transform can be computed.

The harmonic models sadly show a serious shortcoming when they are applied to problems involving potential barriers in that the Wigner transform diverges at low temperature and/or large potential curvature.^3−5,12^ More specifically, it is the off-diagonal part of the density operator matrix elements
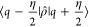
1that becomes meaningless when *ℏ*|Ω|β > π. Here *q* is a coordinate, η is the off-diagonal distance, ρ̂ is the density operator, Ω is the imaginary frequency, and β = 1/(*k*_*B*_*T*) where *T* is the absolute temperature. Under such conditions this matrix element essentially becomes exp(−*αη*^2^) with α < 0; see, e.g., refs (3) and (12).

The momentum part of the Wigner transform is the Fourier transform of the off-diagonal part of the density operator. From the previous paragraph it can be seen that it becomes ill-defined when *ℏ*|Ω|β > π. Although all *moments* of *p* can still be calculated even when α < 0, the Wigner function cannot be used for *sampling* momenta. This is a serious limitation of the harmonic models and ways to modify them have been proposed. For example, Liu and Miller^12^ put forth an ad-hoc extrapolation of the Gaussian momentum exponent α into the forbidden regime, so that a well-defined sampling function exists for all temperatures and frequencies. Poulsen and co-workers have simply chosen to set the momenta to zero and skip the sampling when *ℏ*|Ω|β > π, which is a continuous extrapolation of the *ℏ*|Ω|β ≤ π case; see, e.g., refs (3), (15), and (17).

The harmonic scheme of Poulsen et al.^3,15^ for calculating Wigner transforms is an adoption of the effective frequency theory of Feynman and Kleinert.^18,19^ This scheme has been applied quite successfully to a number of problems in condensed phase involving the quantization of several hundred degrees of freedom. More specifically, the method has been used for calculating dynamic structure factors^15,20−22^ and diffusion coefficients in liquids.^17,23,24^ Despite its relative success, this scheme may be considered as an *illegitimate* extension of the effective frequency theory of Feynman and Kleinert in the sense that the latter is a theory for approximating path integrals involving only *closed* paths, while Poulsen et al.^3^ nevertheless applied it to path integrals which include open paths as well.

Strictly speaking, the extension discussed above is uncontrollable, meaning that divergence problems show up since the effective frequencies are not optimized for open paths. As already explained, these problems appear for barrier potentials at low temperature and this is the reason why the theory has not been applied to reaction rate problems, where a so-called thermalized flux Wigner distributions must be computed.^12^ Reaction rate theory is a central subject in chemistry and it is therefore highly desirable to solve this problem.

The subject of the present paper is to fix the barrier problem by developing an effective frequency theory tailored to path integrals also involving *open* paths. It will be the first rigorous effective frequency model which can calculate off-diagonal elements of the Boltzmann operator and can be combined with general potentials at all temperatures. The theory derived here may be regarded as a first step toward making the effective frequency theory applicable also to reaction rate problems. The derivation is relatively similar to the original one by Feynman and Kleinert;^18,19^ the main difference is the inclusion of open paths, which makes the mathematics slightly more involved.

This paper is structured such that after the above “Introduction” there is a long section on “Methods” where we, based on the Feynman–Kleinert (FK) closed path approach, derive new iterative equations by generalizing the FK approach to include also open paths. This gives us the *Generalized Feynman–Kleinert* (GFK) approach. Thereafter follows a “Results” section before we end with “Discussion and Conclusions”.

## Methods

For simplicity, we formulate the GFK theory in one dimension. The new theory is generalized to several dimensions in the same way as the original Feynman–Kleinert theory; see ref (3). In what follows we, for simplicity, frequently refer to open paths but then actually meaning both open and closed paths unless it is clear from the context that we only consider open paths. We will consider a particle with mass *M* moving in the potential *V*(*x*) at an inverse temperature β = 1/*k*_*B*_*T*.

The open path effective frequency theory is derived by first considering the Feynman path integral for the Boltzmann operator:
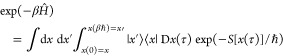
2with
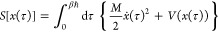
3The centroid of a path, *x*_*c*_, is the average value of the path:
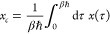
4

We next express the Boltzmann operator using localized operators, Δ̂(*x*_*c*_), whose path integral representations are each restricted to paths with a certain centroid. We write
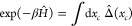
5where
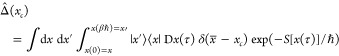
6

We now introduce a new quantity, the *off-diagonal* trace as
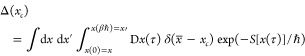
7which differs from a standard trace by also including open paths. Due to the delta-function, it is unitless.^a^ In the following we will use the short-hand notation
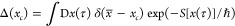
8instead of eq 7. There is no ambiguity in this expression since we consider both closed and open paths. *Thus, in the following ∫*D*x*(τ) *always means integration over closed and open paths.* The idea is now to exploit the introduced locality when calculating the above quantities.

In the high temperature limit, Δ̂(*x*_*c*_) is dominated by paths that only probe a small neighborhood around their centroids. This suggests substituting the actual potential in eq 3 by a *trial* potential of the form
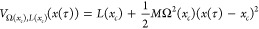
9which is of the same form as used by Feynman and Kleinert. Such a potential should work extremely well at high temperatures. The same form will, however, be used for all temperatures.

For each centroid, two unknown parameters *L*(*x*_*c*_) and Ω^2^(*x*_*c*_) must be determined. As we shall see, their values can respectively be thought of as representing a smeared potential and its second derivative similarly smeared and both evaluated around *x*_*c*_. The smearing width will be derived from the “size” of all paths (open and closed) associated with that centroid.

Once *L*(*x*_*c*_) and Ω^2^(*x*_*c*_) have been found, we can integrate out *all* path variables in eq 6 except the centroid; see below. This makes the effective frequency theory powerful: the original multidimensional path integral becomes one-dimensional and the Wigner transform can be calculated (see eq 48 in ref (3)). Thus, as shown in Appendix A, the Wigner transform of Δ̂(*x*_*c*_) becomes
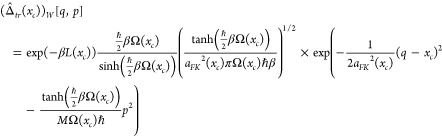
10where *tr* stands for trial and *a*_*FK*_^2^(*x*_*c*_) is given by
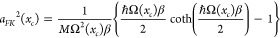
11*a*_*FK*_^2^(*x*_*c*_) comes from Feynman and Kleinert’s original theory and has dimensions length squared. For imaginary Ω(*x*_*c*_) the exponent in the momentum part contains  which diverges when *ℏ*|Ω(*x*_*c*_)|β = π; see ref (3). We point out that eq 10 is the same in the original FK theory and the new generalized effective frequency theory presented here (GFK). The values of Ω(*x*_*c*_) and *L*(*x*_*c*_) as functions of *x*_*c*_ on the other hand differ between the two theories.

Let us now determine *L*(*x*_*c*_) and Ω^2^(*x*_*c*_) for a given centroid *x*_*c*_. The task is to replace *V*(*x*(τ)) in Δ̂(*x*_*c*_) or Δ(*x*_*c*_) with  in the best possible way. After replacement, the off-diagonal trace Δ(*x*_*c*_) is transformed into Δ_*tr*_(*x*_*c*_), where
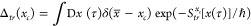
12
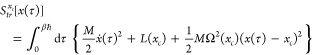
13The off-diagonal trace corresponding to the full Boltzmann operator is approximated as
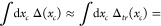
14
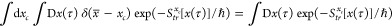
15where we notice that the last equality above is perfectly meaningful since the value of *x*_*c*_ in *S*_*tr*_^*x*_*c*_^[*x*(τ)] is determined by *x*_*c*_ in the path integration *∫*D*x*(τ).

In order to make *∫*d*x*_*c*_ Δ_*tr*_(*x*_*c*_) be as close as possible to *∫*d*x*_*c*_ Δ(*x*_*c*_) we use the Jensen inequality.^25^ We start by writing the exact path integral as
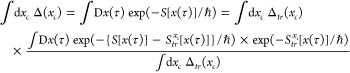
16or
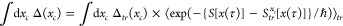
17where the average of a function *f* is calculated by
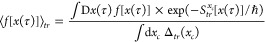
18

The Jensen inequality^25^ rests on the convexity of the exponential function and states that
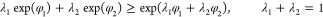
19where φ_1_ and φ_2_ are arbitrary real numbers. Its integral variant is^25^
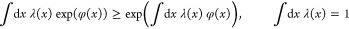
20

Using eq 20 in its multidimensional version with λ[*x*(τ)] = exp(−*S*_*tr*_^*x*_*c*_^[*x*(τ)]/ℏ)/∫d*x*_*c*_ Δ_*tr*_(*x*_*c*_), so that *∫*D*x*(τ) λ[*x*(τ)] = 1, and φ[*x*(τ)] = −(*S*[*x*(τ)] – *S*_*tr*_^*x*_*c*_^[*x*(τ)])/ℏ, we obtain
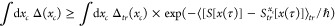
21We will thus seek the values of *L*(*x*_*c*_) and Ω^2^(*x*_*c*_) that maximize the right-hand side of eq 21. For this purpose we derive an expression for Δ_*tr*_(*x*_*c*_) in Appendix A using results from ref (3) and obtain
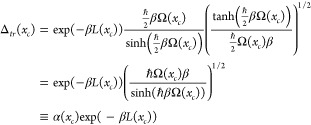
22

Next we wish to evaluate
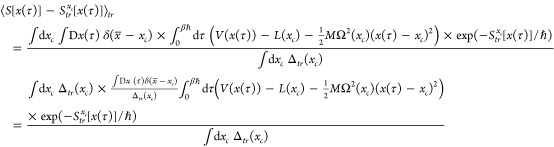
23The quantity
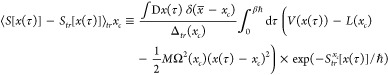
24appearing in eq 23 is also an expectation value, using again the weight-function exp(−*S*_*tr*_^*x*_*c*_^[*x*(τ)]/ℏ) but now evaluated for a *fixed* centroid *x*_*c*_ as opposed to in eq 18 where *x*_*c*_ is integrated over.

We may write
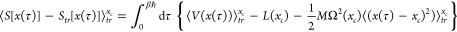
25where we need to calculate the different terms. Let us first look at the average square position found in the last term:
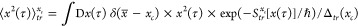
26To evaluate this, we write the paths explicitly using their Fourier representation. As will be seen, once this special term has been found, all other quantities follow straightforwardly. The paths in eq 26 include all closed and open paths *x*(τ) with a common centroid *x*_*c*_.

We wish to find a Fourier representation suitable for describing open paths where *x*_*c*_ occurs explicitly as a Fourier mode. We also need to evaluate the time derivative of *x*(τ) so that we can calculate the kinetic energy of the path. This means that we need to interchange the infinite summation and differentiation operations in our Fourier series expression. This is permissible if *x*(τ) is continuous and *x*′(τ) is integrable.^26^ Thus, we cannot adopt the Fourier series of *x*(τ) directly by regarding it as a βℏ-periodic function, since this function is discontinuous at its open ends. We can instead achieve our requirements by considering a *continuous extension* of *x*(τ) as follows.

We extend our open paths to new ones, *x̃*(τ), defined on the double time interval 0 < τ < 2βℏ. We shall require that our extended paths be identical to the old ones in the original time window: *x̃*(τ) = *x*(τ), 0 < τ < *βℏ* and further that they are symmetric around τ = βℏ, i.e., *x̃*(τ) = *x̃*(2βℏ – τ), which fully determines *x̃*(τ). By this prescription there is a one-to-one correspondence between the *x*(τ) and *x̃*(τ) paths. Further, due to the symmetry, the centroids of *x*(τ) and *x̃*(τ) are identical.

The *x̃*(τ) paths can be defined for all real τ by letting *x̃*(τ) be 2βℏ-periodic. The *x̃*(τ) paths are closed paths, and if we restrict them to be physical, continuous paths, they fulfill all our requirements. We may then write the Fourier series
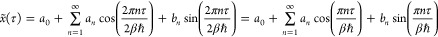
27where *b*_*n*_ = 0 for all *n*, due to the reflection property of *x̃*(τ) around τ = βℏ. If we restrict this Fourier series to 0 < τ < βℏ, we obtain our desired open path representation
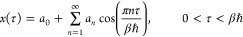
28The basis functions are orthogonal:
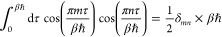
29Clearly, *a*_0_ is the common centroid of *x*(τ) and *x̃*(τ):
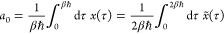
30Likewise, it follows from eq 28 that
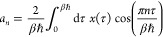
31

In Appendix B, we derive eq 28 again but by using the normal modes of the open polymer. We also show how to set up a general path integral using the path parametrization in eq 28. If we write , the trial action can now be evaluated as (*x*_*c*_ = *a*_0_)
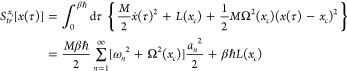
32

Returning now to eq 26, we write^b^
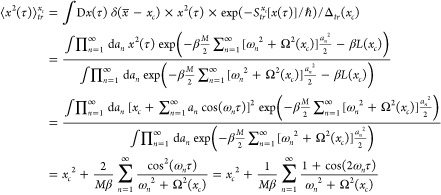
33

Using the result (see page 83 in ref (27))
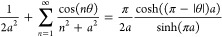
34it is shown in Appendix A that
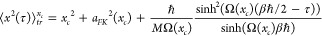
35Here the smearing width *a*_*FK*_^2^(*x*_*c*_), given in eq 11, has appeared. In the closed path theory we would find the simpler result: ⟨*x*^2^(τ)⟩_*tr*_^*x*_*c*_^ = *x*_*c*_^2^ + *a*_*FK*_^2^(*x*_*c*_). Naturally, for closed paths, the quantum fluctuations are independent of τ. According to eq 35, however, for open paths, fluctuations are smallest for intermediate times τ ∼ βℏ/2 where ⟨*x*^2^(τ)⟩_*tr*_^*x*_*c*_^ ∼ *x*_*c*_^2^ + *a*_*FK*_^2^(*x*_*c*_), while they increase exponentially as τ → 0, βℏ.

Since both the second and third terms in eq 35 vanish for high temperatures, it seems reasonable to define a new time-dependent *open-path* smearing width as
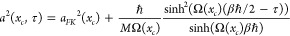
36The last term in this quantity diverges for imaginary Ω(*x*_*c*_) when |Ω(*x*_*c*_)|βℏ = π and becomes infinite when |Ω(*x*_*c*_)|βℏ → π from below. The old smearing width *a*_*FK*_^2^(*x*_*c*_) on the other hand does not diverge until |Ω(*x*_*c*_)|βℏ = 2π. Here we find the reason as to why the new theory is well-behaved and the old not. As |Ω(*x*_*c*_)|βℏ → π from below, the momentum sampling should start to get problematic, but it does not because *at the same time a*^2^(*x*_*c*_, τ) goes to infinity thereby making |Ω(*x*_*c*_)| smaller in size, since the latter is the Hessian of the potential averaged over *a*^2^(*x*_*c*_, τ); see below. The reduced size of |Ω(*x*_*c*_)| pushes |Ω(*x*_*c*_)|βℏ away from π.

Returning to eq 25, we see that we need to find
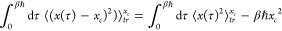
37Inserting eq 35 in eq 37 and integrating over τ, we obtain
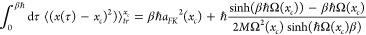
38The average smearing width over the whole open path thus becomes
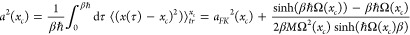
39

We now turn our attention to the term ⟨*V*(*x*(τ))⟩_*tr*_^*x*_*c*_^ in eq 25. An easy way to find it is to consider the Fourier representation of the potential:
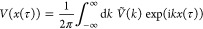
40Using eq 28, we obtain
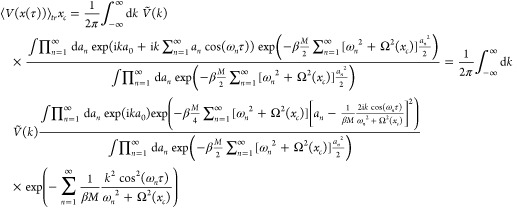
41or (see eq 33)
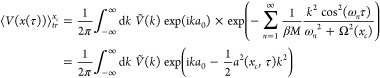
42Inserting the inverse Fourier transform of the potential *V*(*x*)
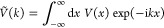
43we obtain
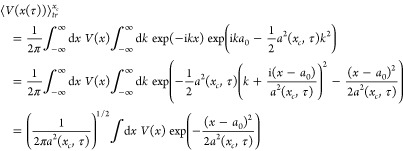
44

This is a Gaussian smearing of the potential around the centroid *x*_*c*_ = *a*_0_. We shall call it *V*_*a*^2^__(*x*_*c*_,τ)_(*x*_*c*_). Hence,
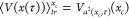
45Equation 25 can now be written
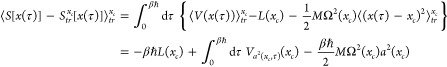
46Putting the pieces together, eq 23 becomes
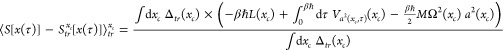
so that eq 21 becomes
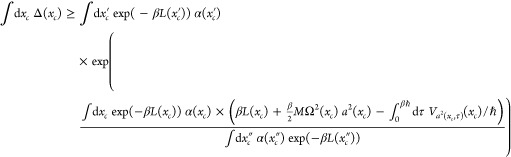
47We call the right-hand side of this inequality *I* = *I*[Ω^2^(*x*_*c*_), *L*(*x*_*c*_)]. Thus, *I* is a functional of the functions Ω^2^(*x*_*c*_) and *L*(*x*_*c*_). We need to make it stationary with respect to variations in *L*(*x*_*c*_) and Ω^2^(*x*_*c*_) for all values of *x*_*c*_. First we will determine the potential *L*(*x*_*c*_) by requiring
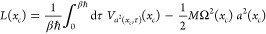
48With this choice, consider the functional variation with respect to *L*(*x*_*c*_). The variation vanishes since
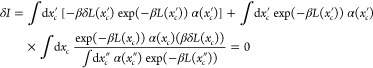
49

Next we turn to the variation of Ω^2^(*x*_*c*_). The right-hand side depends directly on Ω^2^(*x*_*c*_) but also indirectly through *a*^2^(*x*_*c*_), α(*x*_*c*_), and ∫_0_^*βℏ*^d*τ* *V*_*a*^2^(*x*_*c*_,τ)_(*x*_*c*_)/ℏ. Hence there are four contributions to changes in *I* when varying Ω^2^(*x*_*c*_). The functional derivative of *I* with respect to Ω^2^(*x*_*c*_) is
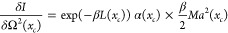
50Next, we calculate the result of variations in Ω^2^(*x*_*c*_) coming from *a*^2^(*x*_*c*_). We write it symbolically
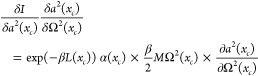
51The third term is
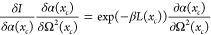
52Finally, the fourth term is
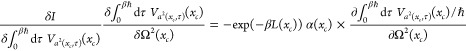
53The sum of these four terms must vanish. As we shall see, we can make the first and third terms sum to zero. Likewise with the second and fourth. Looking at the first pair, we get
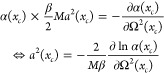
54We already have expressions for *a*^2^(*x*_*c*_) and α(*x*_*c*_) in eqs 39 and 22, respectively. From these we can verify that eq 54 is fulfilled.

Next, let us look at the sum of the second and fourth terms. The equation we get is
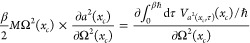
55Using the chain rule, we may write
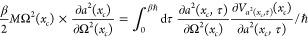
56Since *a*^2^(*x*_*c*_) is the path-averaged smearing width, we have
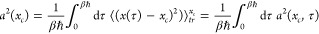
57Thus, we may write
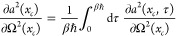
58Equation 56 therefore finally becomes
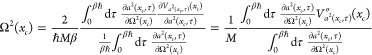
59where we have utilized the equality
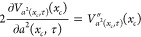
60which follows from manipulations on eq 44.

Equation 59*is our iterative equation which is used together with the expression for a*^2^(*x*_*c*_, τ) *in*eq 36. The iteration starts by first fixing a centroid *x*_*c*_ and choosing a value for Ω^2^(*x*_*c*_). Iteration then goes on until convergence.

In eq 59 we need to evaluate *∂a*^2^(*x*_*c*_, τ)/∂Ω^2^(*x*_*c*_). By differentiating eq 36, the following expression may be found
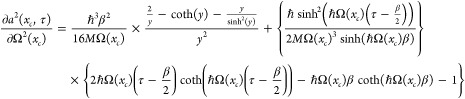
61where we have set ℏΩ(*x*_*c*_)β/2 = *y* for brevity.

For the case of an imaginary frequency, the expressions for *a*^2^(*x*_*c*_, τ), *a*^2^(*x*_*c*_), and *∂a*^2^(*x*_*c*_, τ)/∂Ω^2^(*x*_*c*_) change. By replacing Ω(*x*_*c*_) with *i*|Ω(*x*_*c*_)|, the new expressions become
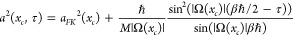
62
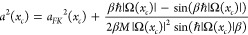
63and
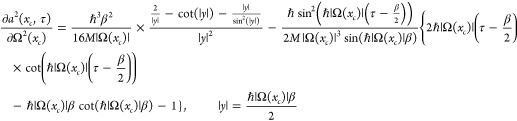
64Here
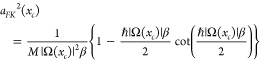
65

## Results

We will apply our new effective frequency theory to two problems, viz. a quartic oscillator and a symmetric double well. The results will be compared with predictions from the original effective frequency theory, classical statistical mechanics as well as accurate calculations. We will use natural units so that ℏ = 1 and we consider a particle with mass *m* = 1.

### The Quartic Oscillator

**Figure 1:**
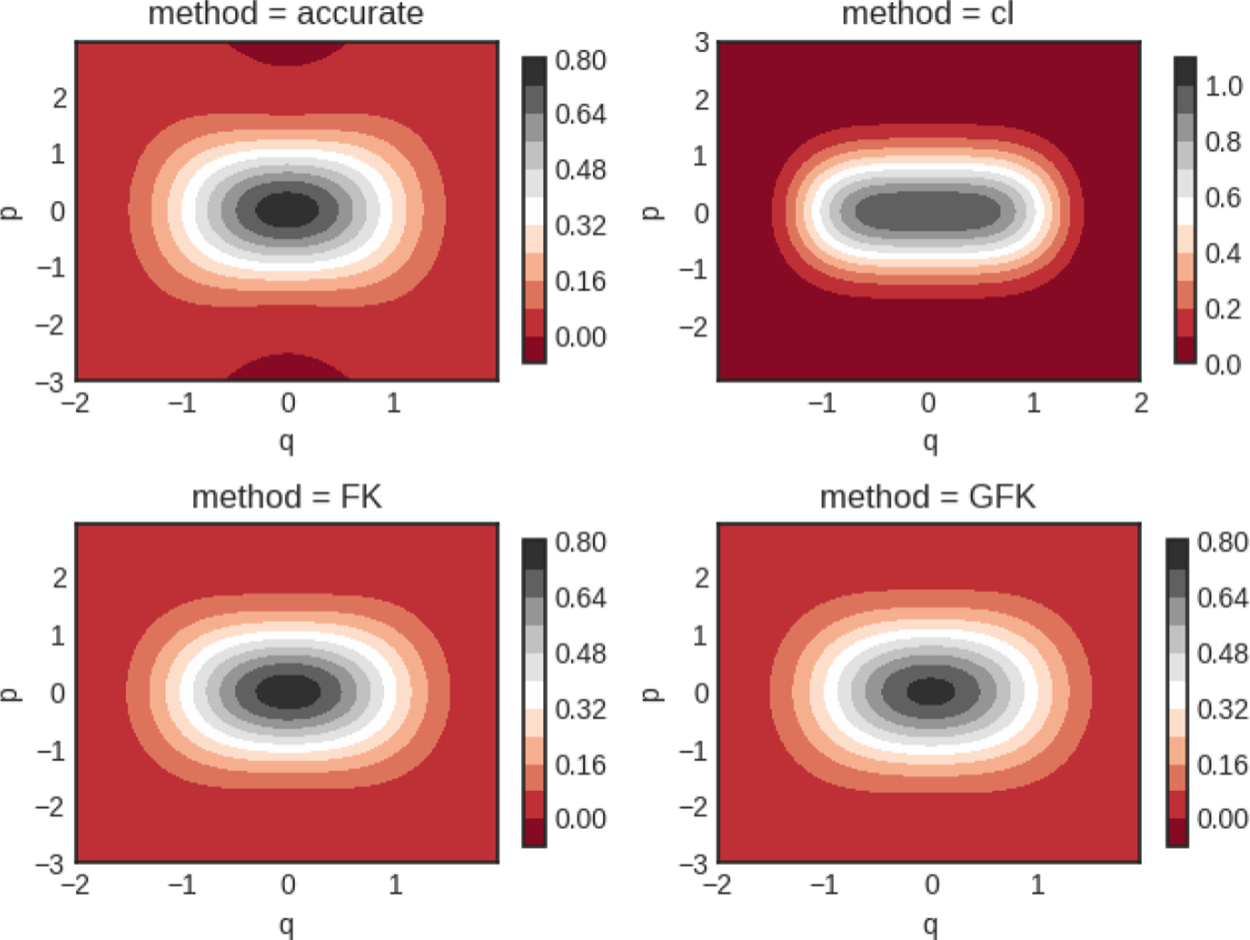
Quartic oscillator Wigner functions for β = 2. Notice the different scale for the classical calculation (cl).

Here we consider a quartic potential *V*(*q*) = *q*^4^/4. In this case we do not encounter any imaginary frequency problem. Hence, the original effective frequency theory is always well-defined. The inverse temperatures that we study are β = 2 and 8. In Figure 1, computed Wigner functions are shown for β = 2 obtained from an accurate calculation, a classical calculation, and the original and new effective frequency models, termed FK and GFK, respectively.

Notice that the Wigner functions are not normalized to unity when integrated. They are Wigner transforms of the Boltzmann operator itself. In Figure 1 good agreement is seen between all four predictions, albeit the classical calculation yields clear deviations from the other results.

**Figure 2:**
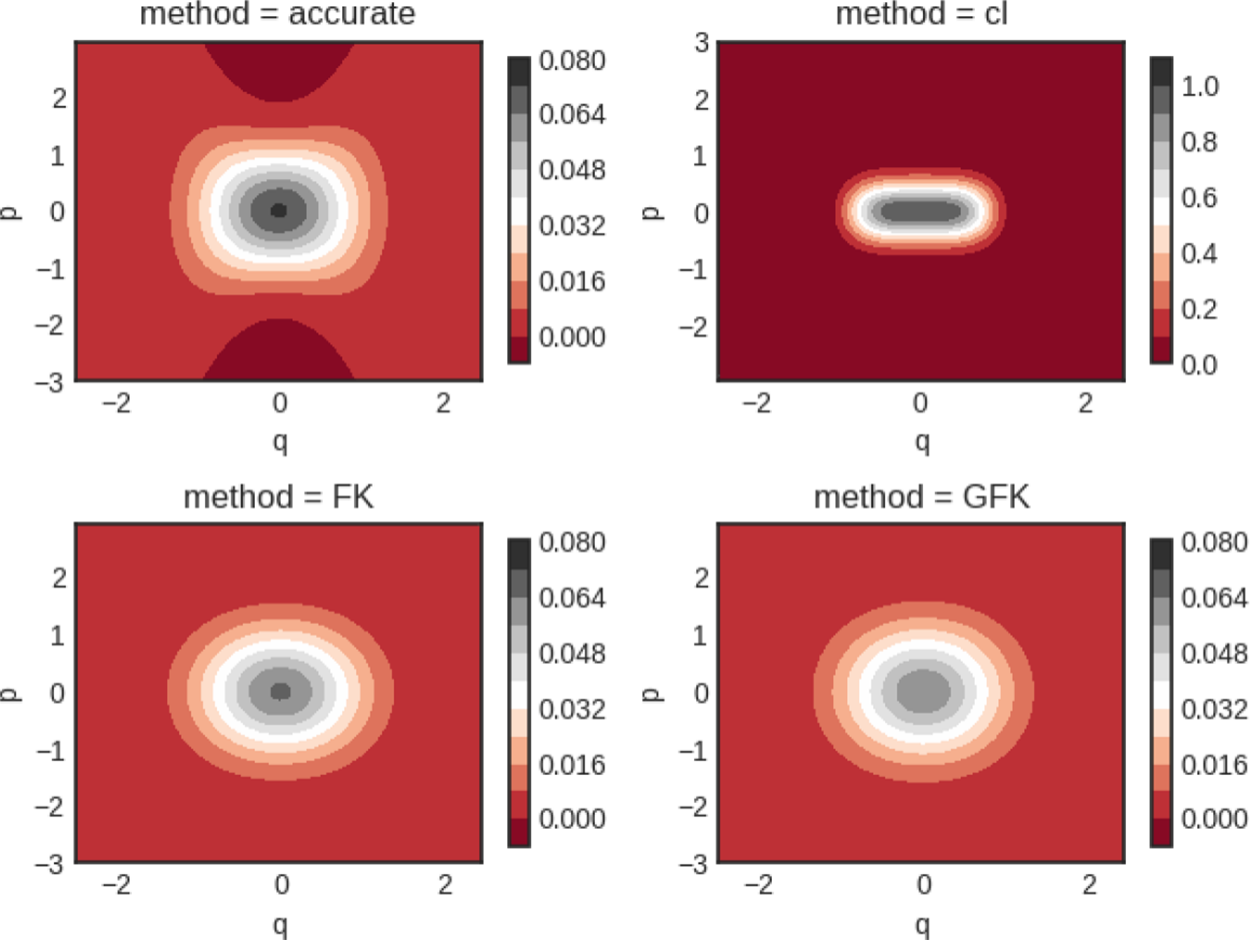
Quartic oscillator Wigner functions for β = 8. Notice the different scale for the classical calculation (cl).

The results for the case β = 8 are shown in Figure 2.

It is clearly seen that the classical result shows large deviation from the other three results. Visually, the two effective frequency theories agree well with each other and also with the accurate quantum result.

In order to make the comparisons between methods more precise, we have adopted the following measure of deviation of an approximate Wigner function, *W*(*q*, *p*), from the accurate Wigner function *W*_*acc*_(*q*, *p*):
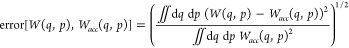
66

In Table 1 such error values are shown for the quartic oscillator at both β = 2 and β = 8.

**Table 1:** Quartic Potential^a^

	FK	GFK	Cl
β = 2	0.02	0.03	0.44
β = 8	0.07	0.10	9.47

a Deviations from the accurate Wigner function are shown for original Feynman–Kleinert effective frequency theory (FK), our generalized effective frequency theory (GFK) and classical theory (Cl).

It is again confirmed that the two effective frequency theories are very close to each other. Moments of the Wigner functions are presented in Table 2.

**Table 2:** Quartic Oscillator^a^

	FK	GFK	accurate	Cl
⟨*q*^2^⟩, β = 2	0.53	0.53	0.53	0.48
⟨*p*^2^⟩, β = 2	0.73	0.79	0.73	0.50
⟨*q*^2^⟩, β = 8	0.45	0.43	0.46	0.24
⟨*p*^2^⟩, β = 8	0.57	0.61	0.56	0.12

a Moments of the Wigner function from original Feynman–Kleinert effective frequency theory (FK), our generalized effective frequency theory (GFK), accurate calculations and classical theory (Cl).

Compared to the accurate calculations and original effective frequency model, the new theory is seen to give slightly larger momenta as the temperature is lowered, while the classical ones are clearly too small.

We will now consider the off-diagonal behavior of the density matrix, i.e., how η behaves in . We define
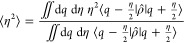
67and show the corresponding values in Table 3 for FK, GFK, and accurate calculations.

**Table 3:** Quartic Oscillator^a^

	FK	GFK	accurate
⟨η^2^⟩ β = 2	1.45	1.32	1.32
⟨η^2^⟩ β = 8	1.77	1.66	1.59

a ⟨η^2^⟩ of the density matrix from the original Feynman–Kleinert effective frequency theory (FK), our generalized effective frequency theory (GFK) and accurate calculations.

The off-diagonal behavior of the density matrix is seen to be better for GFK than for FK.

**Figure 3:**
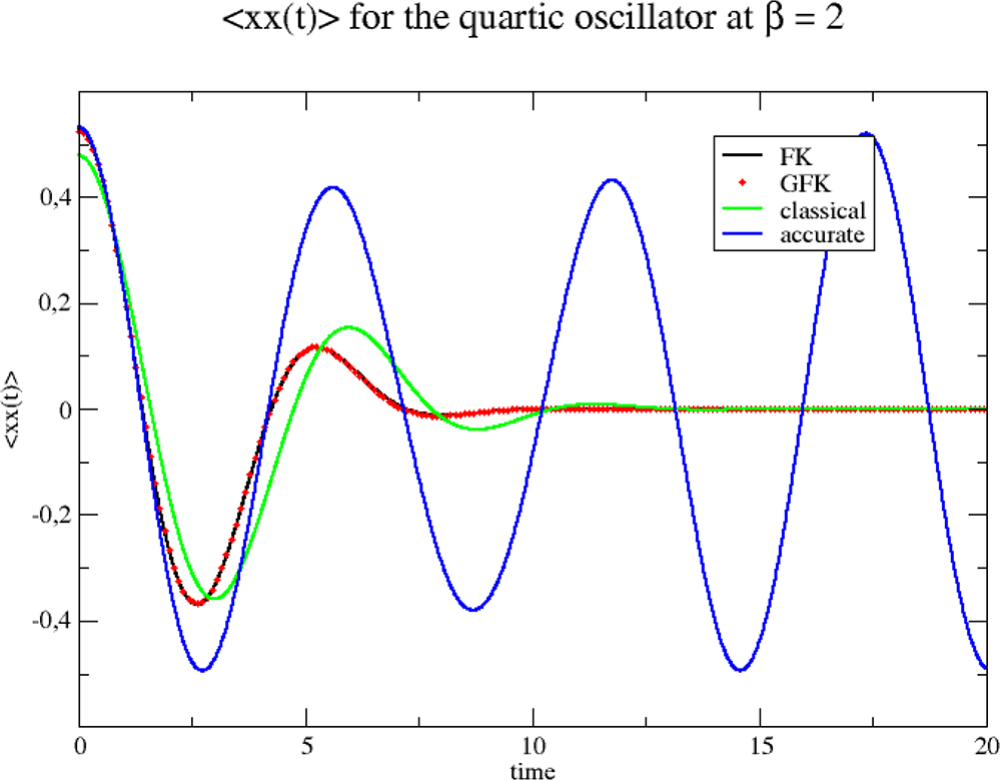
Correlation functions for the quartic oscillator at β = 2. The three Wigner functions used as sampling functions for initiating the classical dynamics are derived from the original Feynman–Kleinert effective frequency theory (FK), our generalized effective frequency theory (GFK), and classical statistical mechanics (classical). Accurate quantum mechanical results are also shown (accurate).

**Figure 4:**
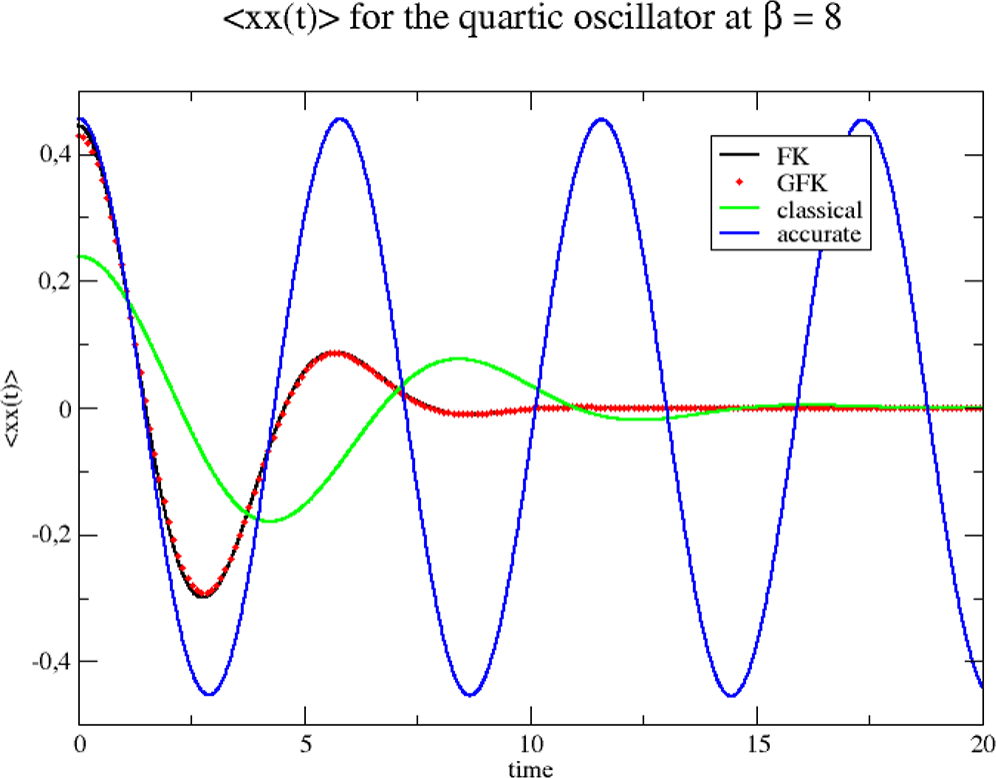
Correlation functions for the quartic oscillator at β = 8. The three Wigner functions used as sampling functions for initiating the classical dynamics are derived from the original Feynman–Kleinert effective frequency theory (FK), our generalized effective frequency theory (GFK), and classical statistical mechanics (classical). Accurate quantum mechanical results are also shown (accurate).

We end this section by showing the position autocorrelation functions, obtained by running classical dynamics using our Wigner functions to sample initial conditions (often referred to as the “Classical Wigner” model). In Figures 3 and 4 we show results for β = 2 and 8, respectively. The figures also contain accurate and purely classical dynamics results. The results from the two effective frequency theories are practically speaking identical.

### Particle in a Double Well

We now turn to a problem with a barrier, namely, the double well, and we choose the potential to be . The double well problem is substantially more challenging than what the quartic potential is. For this potential the momentum part of the Wigner function of the original effective frequency theory becomes ill-defined in the interval β ∈ [5.1, 5.9]. We therefore choose to consider inverse temperatures outside, but reasonably close to, this range. We also consider the results of the so-called local Gaussian approximation (LGA) proposed by Liu and Miller.^12^ This scheme utilizes the following approximation to the Boltzmann operator Wigner transform:
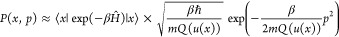
68where *u*(*x*) is the local frequency *u*(*x*)^2^ = *V*^″^(*x*)/*m* and *Q*(*u*(*x*)) is given by
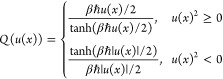
69

**Figure 5:**
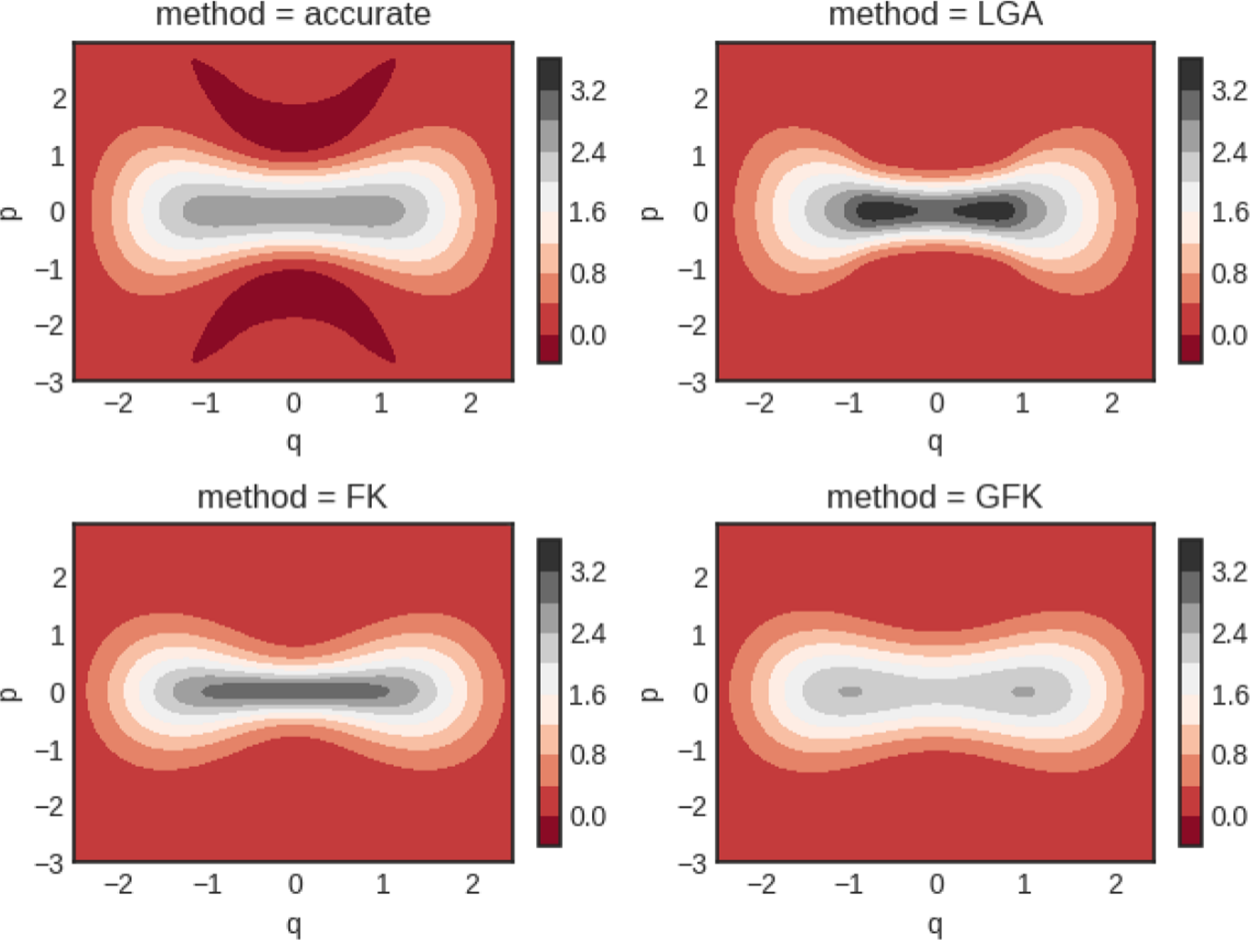
Double well Wigner functions for β = 4. Although not shown, the classical Wigner function has a peak value of around 13.5.

**Figure 6:**
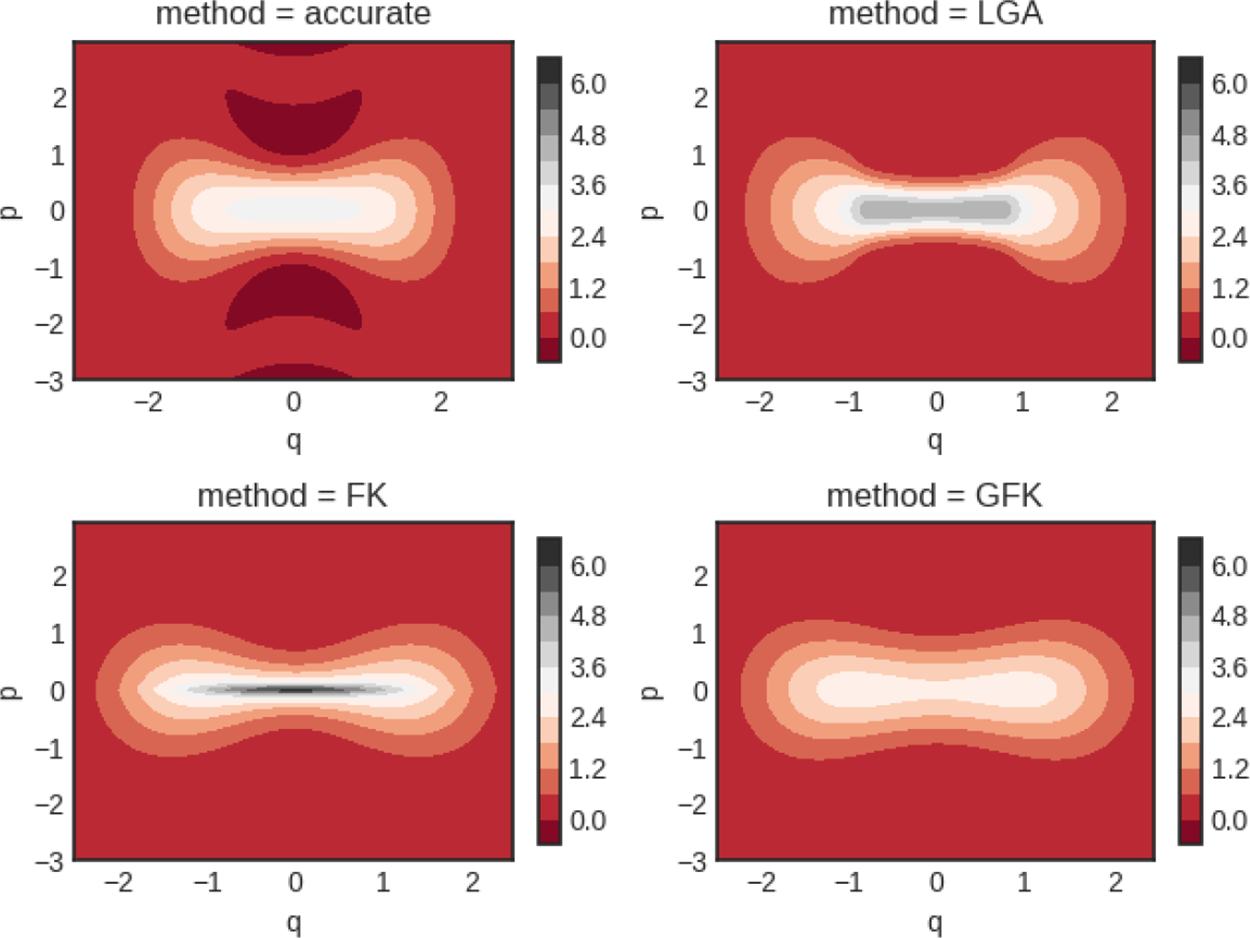
Double well Wigner functions for β = 5. Although not shown, the classical Wigner function has a peak value of around 25.

**Figure 7:**
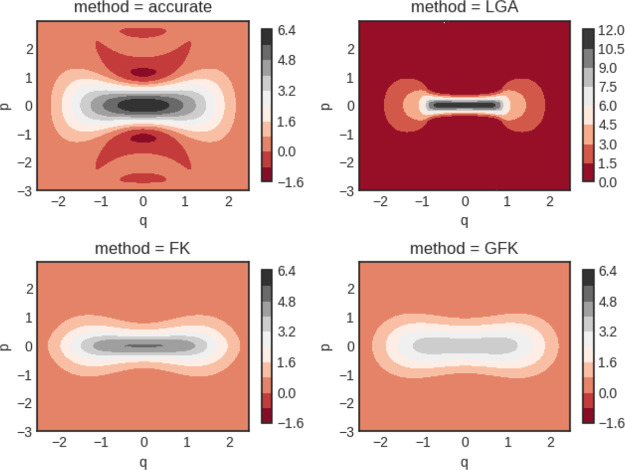
Double well Wigner functions for β = 8. Notice the different scale for the LGA calculation. Although not shown, the classical Wigner function has a peak value of around 150.

Equation 68 is always well-defined. Also, the phase-space trace of *P*(*x*, *p*) is exact so the Wigner function is correctly normalized. In Figures 5–7 we show the Wigner distributions for β = 4, 5, and 8, respectively, for the four methods that we compare, namely, accurate, FK, GFK, and LGA.

For β = 4, it is seen that the Wigner function derived from the original Feynman–Kleinert theory possesses a too narrow momentum distribution near the barrier top in comparison to the accurate result. The situation is similar for the LGA Wigner function. Both of these functions also have a too large maximum value of *P*(*x*, *p*). In comparison, the GFK function behaves better. In the case of β = 5, where ℏ|Ω(*x*_*c*_)|β is closer to π, the same observations hold but they are more accentuated. For β = 8, the LGA Wigner function decays very rapidly along the *p* axis and deviates substantially from the accurate one.

It is hard to judge how close the approximate Wigner functions are to the accurate one. The comparison is further complicated by the fact that only the exact function can be negative. Again we may turn to our error estimate. In Table 4, we present error values calculated using eq 66.

**Table 4:** Double Well Results^a^

	FK	GFK	LGA	Cl
β = 4	0.14	0.14	0.16	2.0
β = 5	0.26	0.18	0.25	3.4
β = 8	0.28	0.35	0.52	12
β = 10	0.35	0.42	0.67	29

a Deviations from accurate Wigner function results calculated from eq 66. Results are shown for the original Feynman–Kleinert effective frequency theory (FK), our generalized effective frequency theory (GFK), local Gaussian approximation (LGA),^12^ and purely classical theory (Cl).

Again we see that the results for the two effective frequency theories are quite close to each other and much better than the classical results. Note that for β = 5, as opposed to for the other β-values, FK is sligthly worse than GFK. This is because β = 5 is quite close to the β-range where FK becomes ill-defined. The LGA approximation is seen to perform slightly worse than the two effective frequency theories.

We may also consider moments of the Wigner function; see Table 5.

**Table 5:** Double Well Results for Moments of the Wigner Functions^a^

	FK	GFK	LGA	accurate	Cl
⟨*q*^2^⟩, β = 4	1.71	1.66	1.71	1.71	2.18
⟨*p*^2^⟩, β = 4	0.48	0.54	0.48	0.49	0.25
⟨*q*^2^⟩, β = 5	1.65	1.59	1.65	1.65	2.23
⟨*p*^2^⟩, β = 5	0.44	0.51	0.44	0.44	0.20
⟨*q*^2^⟩, β = 8	1.53	1.48	1.55	1.55	2.34
⟨*p*^2^⟩, β = 8	0.40	0.46	0.39	0.36	0.12
⟨*q*^2^⟩, β = 10	1.47	1.43	1.52	1.52	2.38
⟨*p*^2^⟩, β = 10	0.39	0.44	0.37	0.33	0.10

a Results are shown for the original Feynman–Kleinert effective frequency theory (FK), our generalized effective frequency theory (GFK), local Gaussian approximation (LGA),^12^ accurate calculations, and classical theory (Cl).

The original Feynman–Kleinert effective frequency theory is seen to give results quite close to the accurate ones. Our new method systematically slightly overestimates the momentum of the system and similarly slightly underestimates ⟨*q*^2^⟩, while the classical results are substantially worse. The LGA approximation is clearly seen to work well. In fact, the LGA model predicts the most correct moments, being slightly closer to the accurate ones than the original FK model.

In Table 6 we show the average value of η^2^ for the double well at various temperatures. When β is near the interval [5.1, 5.9], we see the divergence behavior in ⟨η^2^⟩ as obtained from FK theory. Also, the LGA approximation performs poorly: It predicts too large values of ⟨η^2^⟩ which is equivalent to a very slowly decaying density matrix in its off-diagonal direction. The GFK model predicts values of ⟨η^2^⟩ being in much better agreement with the accurate values. Thus, the GFK method outperforms the LGA and original effective frequency model when it comes to predicting the off-diagonal behavior of the density matrix.

**Table 6:** Double Well Results for ⟨η^2^⟩ of the Density Matrix^a^

	FK	GFK	LGA	accurate
⟨η^2^⟩, β = 4	6.72	2.36	3.68	2.63
⟨η^2^⟩, β = 5	133	2.53	5.14	2.97
⟨η^2^⟩, β = 6	106	2.63	6.97	3.22
⟨η^2^⟩, β = 8	7.13	2.76	11.79	3.56

a Results are shown from original Feynman–Kleinert effective frequency theory (FK), our generalized effective frequency theory (GFK), local Gaussian approximation (LGA),^12^ and accurate calculation.

**Figure 8:**
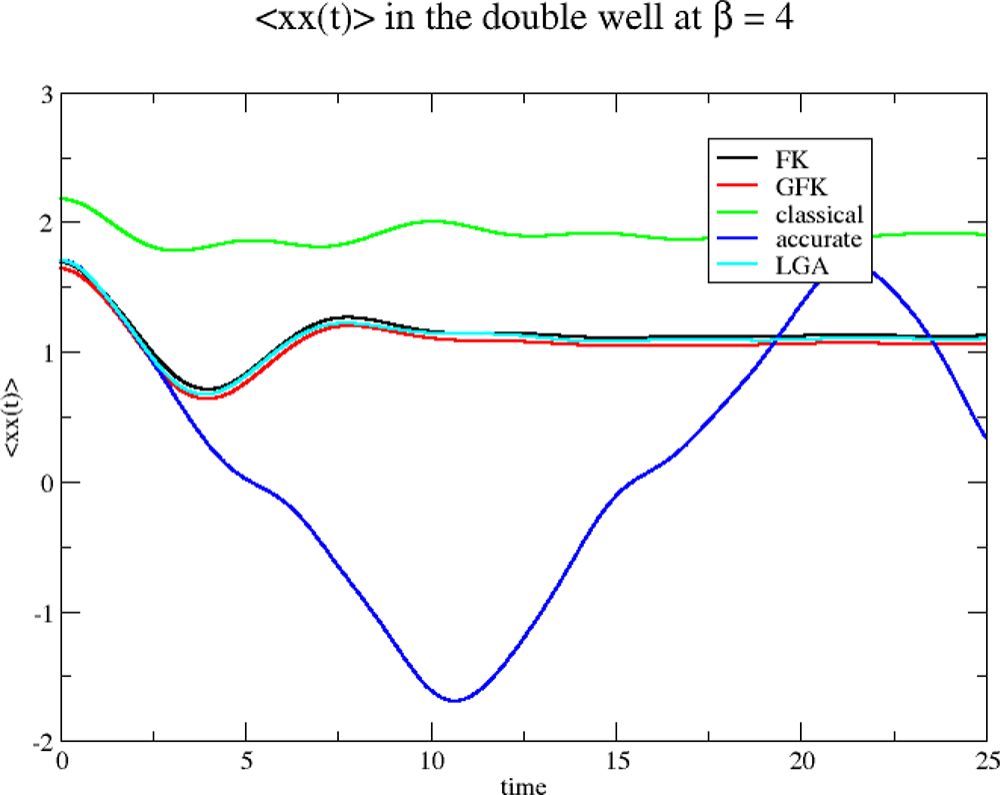
Position autocorrelation functions for the double well at β = 4. The four Wigner functions used as sampling functions for initiating the classical dynamics are derived from the original Feynman–Kleinert effective frequency theory (FK), our generalized effective frequency theory (GFK), local Gaussian approximation (LGA), and classical statistical mechanics (classical). Accurate quantum mechanical results are also shown (accurate).

**Figure 9:**
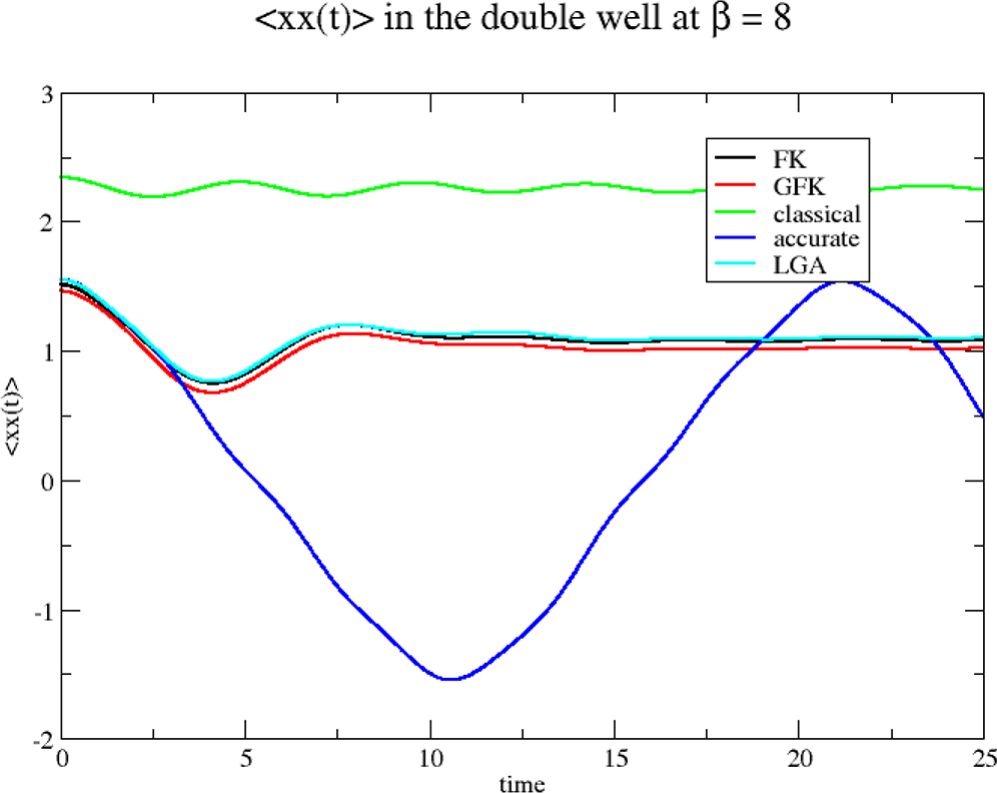
Position autocorrelation functions for the double well at β = 8. The four Wigner functions used as sampling functions for initiating the classical dynamics are derived from the original Feynman–Kleinert effective frequency theory (FK), our generalized effective frequency theory (GFK), local Gaussian approximation (LGA), and classical statistical mechanics (classical). Accurate quantum mechanical results are also shown (accurate).

As in the previous section, we show the position autocorrelation functions, obtained from the Classical Wigner model. This is done in Figures 8 and 9 for β = 4 and 8, respectively.

The results for the two effective frequency theories and the LGA result agree quite well with each other. They are all clearly better than the classical results. We conclude that it does not matter which of the three theories you adopt for the position autocorrelation functions. Due to the Classical Wigner model itself, the results obtained are essentially equally far away from the accurate result.

## Discussion and Conclusions

A new effective frequency model for imaginary time path integrals has been proposed. Since the new theory is a generalization of the Feynman–Kleinert (FK) theory in that it also includes open paths, we term it the Generalized Feynman–Kleinert theory (GFK). More specifically, GFK “trains” the effective frequency trial action also on open paths as opposed to the original model which was developed for closed paths only. This removed the divergence problem inherent in the original Feynman–Kleinert theory, when obtaining the Wigner functions for barrier problems. The inclusion of open paths in the variational procedure was shown to lead to a more accurate off-diagonal behavior of the density matrix. The GFK theory is, however, not generally more accurate than the original closed-path model of Feynman–Kleinert. On the contrary, it was seen to provide quadratic moments that are slightly worse than those predicted from the original FK model (and LGA model as well).

What are the reasons for the observations mentioned just above? We first point out that the two effective frequency theories utilize the same trial action. GFK is thus not more elaborate. It, however, “trains” its trial action on a larger class of paths. It is therefore not surprising that when comparing moments such as, e.g., ⟨*x*^2^⟩ and ⟨*p*^2^⟩, which may be calculated using only *closed* paths, the FK theory is more precise, since it is optimized for precisely such paths. The present study, however, suggests that when calculating such quantities the differences between the two models are very small. On the other hand, in studying quantities depending on the off-diagonal behavior of the density matrix, GFK outperforms FK for temperatures close to where the FK theory leads to divergence issues.

The results of this paper, suggest that the GFK theory provides a “Jack of all trades” Wigner transform approach, since its density matrix and Wigner transform plots are always consistent (but not always the most accurate). This should be contrasted to the FK theory with its divergence issues and the LGA model which predicts an errorneous rapid decay of its double well Wigner function along the momentum axis.

**Figure 10:**
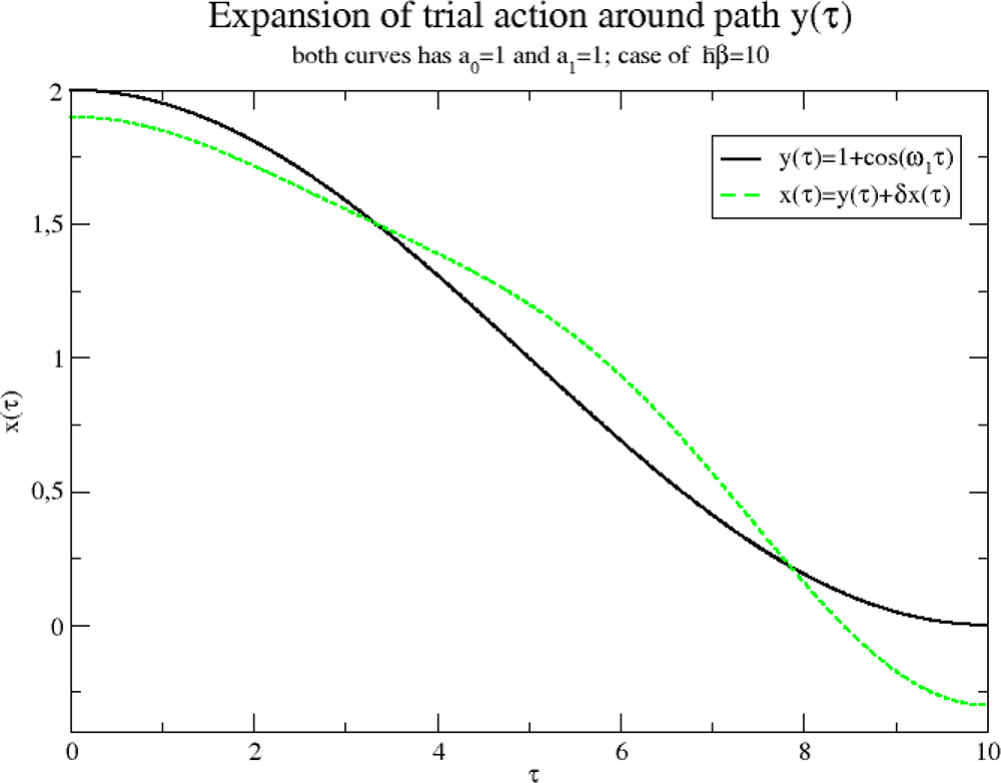
Trial action *x*(τ) expanded around a path *y*(τ).

We end by pointing out immediate possible generalizations. Instead of including just the centroid, we could include additional Fourier modes in the anharmonic part of the trial action. For instance, we could choose to use the two lowest modes *a*_0_ and *a*_1_, which turns the trial action into
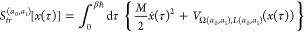
70where
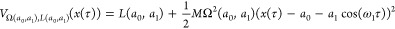
71This is a generalization of eq 13. Thus, the action is now expanded harmonically around a *time-dependent* path *y*(τ) = *a*_0_ + *a*_1_ cos(ω_1_τ); see Figure 10.

All developments proceed essentially as before and the required expressions can be calculated with the path integral technique outlined in Appendix B.

By proceeding as just outlined but including more and more modes, the trial action approaches the exact action. In multidimensional applications, we could apply this multimode theory to the most important degrees of freedom, but use the single mode version detailed in this paper for the other degrees of freedom. Further, we could apply similar ideas as presented here for developing trial actions for path integrals involving thermalized flux operators. In this case, besides the centroid, we would impose further constraints on the open paths by requiring the paths to pass the dividing surface at τ = βℏ/2; see, e.g., refs (4) and (28). As shown in the Supporting Information, such further restrictions can be implemented by inserting delta functions into the off-diagonal path integral.

## Appendix A

### Miscellaneous Results

The purpose of this appendix is to derive eqs 10, 22, and 35. We first derive eq 10. We start from eq 10 in ref (23) which gives the Feynman–Kleinert approximation to the Wigner transform of the Boltzmann operator. It reads

72

where α = α(*x*_*c*_) is related to the Feynman–Kleinert smearing width by α = 2*M*Ω(*x*_*c*_)*a*_*FK*_^2^(*x*_*c*_)/ℏ. The centroid potential is .

Let Δ̂_*tr*_(*x*_*c*_) denote the operator you get from Δ̂(*x*_*c*_) by replacing the true potential by the Feynman–Kleinert trial potential in eq 9. From the definition of Δ̂(*x*_*c*_) in eq 8, it follows that by adopting the FK trial potential

73

and so by comparing with eq 72 we get

74

which is eq 10.

We next derive eq 22. By performing an inverse Wigner transform on eq 74, we get

75

The off-diagonal trace is just integrating ⟨*x*′|Δ̂_*tr*_(*x*_*c*_)|*x*⟩ over both *x* and *x*′. The result is eq 22.

Finally we derive eq 35. By combining eqs 33 and 34, we obtain

76

Using coth(*x*) =  coth(*x*/2) – 1/ sinh(*x*), we rewrite the third line so that

77

Here the old smearing width from the original Feynman–Kleinert theory has appeared. It is

and fulfils ⟨*x*^2^(τ)⟩_*tr*_ = *a*_*FK*_^2^(*x*_*c*_) + *x*_*c*_^2^ if only closed paths are considered. The first part of eq 77 can be simplified by using cosh(2*x*) = 2 cosh^2^(*x*) – 1 = 1 + 2 sinh^2^(*x*). We then have

78

or

79

which is eq 35.

## Appendix B

### Path Integral Using Open Path Fourier Modes

Here we show how to write the path integral representation of the Boltzmann operator using Fourier modes. Normally, this is done assuming closed paths, using the so-called Matsubara frequency representation.^19^ Here, we do it for open paths, which to our knowledge is much less explored. As an example, we consider the calculation of the off-diagonal trace Δ = *∫*d*x*_*c*_ Δ(*x*_*c*_) for a one-dimensional particle with mass *M* moving in a potential *V*(*x*) at an inverse temperature β = 1/*k*_*B*_*T*. In Cartesian coordinates, using *N* beads, we have

80

Using the high temperature approximation (writing β′ = β/(*N* – 1))

81

we obtain

82

where we have introduced some short-hand notation: *x⃗*_*N*_ = (*x*_1_, ..., *x*_*N*_) and . We next introduce the normal modes of the kinetic energy, which is written in the form ∑_*k*=1_^*N*–1^(*x*_*k*+1_ – *x*_*k*_)^2^.^29^ The *j*th normal mode is

83

where the orthonormal *N* × *N* matrix *Q* has components

84

The eigenvalue of ν_*j*_ is

85

It follows that

86

By expressing eq 82 in terms of normal modes, it becomes

87

We have written *ν⃗*_*N*_ = (ν_0_, ..., ν_*N*–1_) and utilized that ∫d*ν⃗*_*N*_ = ∫d*x⃗*_*N*_ since the determinant of the Jacobian of the mapping between *ν⃗*_*N*_ and *x⃗*_*N*_ is unity. For mathematical convenience we will define *η⃗*_*N*_ = (1/*N*)^1/2^*ν⃗*_*N*_. It follows that η_0_ is the centroid of the path. We can write

88

One final coordinate transformation is needed. We set

89

and

90

Again we write *a⃗*_*N*_ = (*a*_0_,..,*a*_*N*–1_). Equation 88 tranforms into

91

Using the identity (see proof at the end of this section)

92

Equation 91 can be simplified to

93

where , *j* = 0, ..., *N* – 1. Equation 93 is the Fourier representation of the path integral.

We next consider the equation for *a*_*j*_ (*j* ≠ 0):

94

Fix a value of *j*. If *N* is large in eq 94, this expression may be written (use sin(*x*)/*x* → 1, *x* → 0)

95

We have discretized the time axis as , *k* = 1, ..., *N*. For *j* = 0, we obtain similarly

96

We recognize eqs 95 and 96 as eq 31 and eq 30, respectively. Thus, we have rederived the Fourier representation of *x*(τ).

We may also consider the expression for *x*_*k*_ in terms of the normal modes in the limit *N* → *∞*. Again, let , *k* = 1, ..., *N*. From eq 86 we get

97

To proceed, we need to show that γ(*j*, *N*) ≡  can be set equal to unity for large *N*. This is correct as long as *j* ≪ *N*. At first sight, this is not possible since *j* runs all the way up to *N* – 1. However, all realistic paths *x*(τ) that contribute to the path integral have a frequency cut-off *j* ≤ *j*_*max*_ which results from the kinetic energy part of the path integral. Therefore, if *j* is so big that γ(*j*, *N*) is not unity, then *j* will by far exceed *j*_*max*_ if *N* is big enough. Thus, if *j* > *j*_*max*_ then only *a*_*j*_ = 0 contributes to the path integral and it will not matter if we put γ(*j*, *N*) = 1 in eq 97.

If *N* becomes large in eq 97 and *k* likewise so that  is constant, then we get the following expression for *x*(τ_*k*_):

98

This is eq 28.

We finally show how to express the potential energy part of the path integral in terms of Fourier modes as done in eq 93. The part is

99

This rewriting should be acceptable for large *N*. When *N* → *∞*, this becomes

100

Then we may finally write the total path integral in eq 93 as

101

where we have taken the limit *N* → *∞*.

Equation 101 is the central result of this appendix. If we restrict the centroid *a*_0_ to be *a*_0_ = *x*_*c*_ and consider a potential , then we may show that eq 101 rederives the result in eq 22.

We remember that eq 101 is the Boltzmann path integral over *all* open paths. If we want to calculate matrix elements such as, e.g., ⟨*x*| exp(−*βĤ*)|*y*⟩ we can apply eq 101 if we add two extra integrations δ(*y* – *x*(0)) = *∫*d*θ*_1_ exp(i*θ*_1_(*y* – *x*(0)))/2π = *∫*d*θ*_1_ exp(i*θ*_1_(*y* – *a*_0_ – *a*_1_ – ...))/2π and δ(*x* – *x*(βℏ)) = *∫*d*θ*_2_ exp(i*θ*_2_(*x* – *a*_0_ + *a*_1_ – *a*_2_ + ...))/2π. In this Gaussian integral, we first integrate out all (*a*_0_, *a*_1_, ...), followed by integrating out θ_1_ and θ_2_. In this way, also eq 75 can be derived by simply fixing the centroid *a*_0_ to be *a*_0_ = *x*_*c*_.

Let us finally return to the identity in eq 92. To prove it, we start with the result 1.392.1 in ref (30)

102

Letting *x* → 0, we obtain

103

Next, let *n* = 2*m* be even. Observe that the arguments of the sine functions go through half the unit circle, symmetrically around π/2. By using , we may limit the sine arguments to the first quarter of the unit circle only, instead squaring the result. Thus

104

or

105

which was to be proved.

## Supporting Information Available

Calculation of matrix elements of the Boltzmann operator using Fourier Modes - the harmonic oscillator case (PDF)

## Footnotes

a We notice that by adopting the standard *N*-bead time-sliced path integral approximation to Δ(*x*_*c*_), we obtain the classical partition function for an *N*-bead polymer which is held together by harmonic springs with force constants *k* = *M*(*N* – 1)^2^/(*βℏ*)^2^ and moves in the external potential *V*(*x*) at inverse temperature β/(*N* – 1). Its center of mass is fixed at *x*_*c*_. See Appendix B for the details of this derivation.

b When evaluating eq 33, the determinant of the Jacobian which is associated with the mapping from Cartesian coordinates to Fourier modes is not needed, since we are calculating the ratio of two path integrals.

## References

[ref1] Wigner E. On the Quantum Correction For Thermodynamic Equilibrium. Phys. Rev. 1932, 40, 749 10.1103/PhysRev.40.749.

[ref2] Zachos C. K., Fairlie D. B., Curtright T. L., Eds. Quantum Mechanics in Phase Space; World Scientific, 2005.

[ref3] Poulsen J. A.; Nyman G.; Rossky P. J. Practical evaluation of condensed phase quantum correlation functions: A Feynman–Kleinert variational linearized path integral method. J. Chem. Phys. 2003, 119, 12179–12193. 10.1063/1.1626631.

[ref4] Wang H.; Sun X.; Miller W. H. Semiclassical approximations for the calculation of thermal rate constants for chemical reactions in complex molecular systems. J. Chem. Phys. 1998, 108, 9726–9736. 10.1063/1.476447.

[ref5] Shi Q.; Geva E. Semiclassical Theory of Vibrational Energy Relaxation in the Condensed Phase. J. Phys. Chem. A 2003, 107, 9059–9069. 10.1021/jp030497+.

[ref6] Bose A.; Makri N. Wigner Distribution by Adiabatic Switching in Normal Mode or Cartesian Coordinates and Molecular Applications. J. Chem. Theory Comput. 2018, 14, 5446–5458. 10.1021/acs.jctc.8b00179. 30346773

[ref7] Plé T.; Huppert S.; Finocchi F.; Depondt P.; Bonella S. Sampling the thermal Wigner density via a generalized Langevin dynamics. J. Chem. Phys. 2019, 151, 114114 10.1063/1.5099246. 31542021

[ref8] Marinica D. C.; Gaigeot M.-P.; Borgis D. Generating approximate Wigner distributions using Gaussian phase packets propagation in imaginary time. Chem. Phys. Lett. 2006, 423, 390–394. 10.1016/j.cplett.2006.04.007.

[ref9] Smith K. K. G.; Poulsen J.; Nyman G.; Rossky P. J. A new class of ensemble conserving algorithms for approximate quantum dynamics: Theoretical formulation and model problems. J. Chem. Phys. 2015, 142, 244112 10.1063/1.4922887. 26133415

[ref10] Svensson K.-M.; Poulsen J.; Nyman G. Classical Wigner model based on a Feynman path integral open polymer. J. Chem. Phys. 2020, 152, 094111 10.1063/1.5126183. 33480737

[ref11] Weinbub J.; Ferry D. K. Recent advances in Wigner function approaches. Appl. Phys. Rev. 2018, 5, 041104 10.1063/1.5046663.

[ref12] Liu J.; Miller W. H. A simple model for the treatment of imaginary frequencies in chemical reaction rates and molecular liquids. J. Chem. Phys. 2009, 131, 074113 10.1063/1.3202438. 19708738

[ref13] Liu J.; Miller W. H. Using the thermal Gaussian approximation for the Boltzmann operator in semiclassical initial value time correlation functions. J. Chem. Phys. 2006, 125, 224104 10.1063/1.2395941. 17176131

[ref14] Liu J.; Miller W. H. An approach for generating trajectory-based dynamics which conserves the canonical distribution in the phase space formulation of quantum mechanics. II. Thermal correlation functions. J. Chem. Phys. 2011, 134, 104102 10.1063/1.3555274. 21405151

[ref15] Poulsen J. A.; Nyman G.; Rossky P. J. Feynman-Kleinert Linearized Path Integral (FK-LPI) Algorithms for Quantum Molecular Dynamics, with Application to Water and He(4). J. Chem. Theory Comput. 2006, 2, 1482–1491. 10.1021/ct600167s. 26627018

[ref16] Liu J. Recent advances in the linearized semiclassical initial value representation/classical Wigner model for the thermal correlation function. Int. J. Quantum Chem. 2015, 115, 657–670. 10.1002/qua.24872.

[ref17] Poulsen J. A.; Nyman G.; Rossky P. J. Static and dynamic quantum effects in molecular liquids: A linearized path integral description of water. Proc. Natl. Acad. Sci. U. S. A. 2005, 102, 6709–6714. 10.1073/pnas.0408647102. 15860585 PMC1100757

[ref18] Feynman R. P.; Kleinert H. Effective classical partition functions. Phys. Rev. A: At., Mol., Opt. Phys. 1986, 34, 5080–5084. 10.1103/PhysRevA.34.5080. 9897894

[ref19] Kleinert H. Path Integrals in Quantum Mechanics, Statistics, Polymer Physics, and Financial Markets, 5th ed.; World Scientific: Singapore, 2009.

[ref20] Poulsen J. A.; Nyman G.; Rossky P. J. Determination of the Van Hove Spectrum of liquid He(4): An application of the Feynman-Kleinert Linearized Path Integral Methodology. J. Phys. Chem. A 2004, 108, 8743–8751. 10.1021/jp049281d.

[ref21] Scheers J.; Poulsen J. A.; Nyman G.; Rossky P. J. Quantum density fluctuations in liquid neon from linearized path-integral calculations. Phys. Rev. B: Condens. Matter Mater. Phys. 2007, 75, 224505 10.1103/PhysRevB.75.224505.

[ref22] Smith K. K. G.; Poulsen J.; Nyman G.; Cunsolo A.; Rossky P. J. Application of a new ensemble conserving quantum dynamics simulation algorithm to liquid para-hydrogen and ortho-deuterium. J. Chem. Phys. 2015, 142, 244113 10.1063/1.4922888. 26133416

[ref23] Poulsen J. A.; Nyman G.; Rossky P. J. Quantum Diffusion in Liquid Para-hydrogen: An Application of the Feynman-Kleinert Linearized Path Integral Approximation. J. Phys. Chem. B 2004, 108, 19799–19808. 10.1021/jp040425y.

[ref24] Hone T. D.; Poulsen J. A.; Rossky P. J.; Manolopoulos D. E. Comparison of Approximate Quantum Simulation Methods Applied to Normal Liquid Helium at 4 K. J. Phys. Chem. B 2008, 112, 294–300. 10.1021/jp075022n. 18027920

[ref25] Cover T. M.; Joy T. A. Elements of Information Theory, 2nd ed.; Wiley-Interscience, 2006.

[ref26] Taylor A. E. Differentiation of Fourier Series and Integrals. American Mathematical Monthly 1944, 51, 19–25. 10.1080/00029890.1944.11990159.

[ref27] Feynman R. P. Statistical Mechanics: A set of Lectures; Addison-Wesley: Boston, MA, 1998.

[ref28] Poulsen J.; Huaqing L.; Nyman G. Classical Wigner method with an effective quantum force: Application to reaction rates. J. Chem. Phys. 2009, 131, 024117 10.1063/1.3167299. 19603980

[ref29] Verdier P. H. Monte Carlo Studies of Lattice-Model Polymer Chains. I. Correlation Functions in the Statistical-Bead Model. J. Chem. Phys. 1966, 45, 2118–2121. 10.1063/1.1727896.

[ref30] Gradshteyn I. S., Ryzhik I. M., Eds. Table of Integrals, Series, and Product, 7th ed.; Elsevier/Academic Press: Amsterdam, 2007.

