# Supplementary material for: A Divergence-Free Wigner Transform of the Boltzmann Operator Based on an Effective Frequency Theory
Source: J Phys Chem A. 2021 Oct 12;125(41):9209–25. doi: 10.1021/acs.jpca.1c05860 (PMC8543443; doi:10.1021/acs.jpca.1c05860)
Supplement: Supplementary file 1 — jp1c05860_si_001.pdf [file jp1c05860_si_001.pdf]

Supporting Information for:

A Divergence-free Wigner Transform of the Boltzmann Operator Based on an Effective Frequency Theory

Jens Aage Poulsen and Gunnar Nyman*

Department of Chemistry and Molecular Biology, University of Gothenburg, SE 405 30, Gothenburg, Sweden

E-mail: gunnar.nyman@gu.se

Harmonic oscillator matrix elements.

In Appendix B of the main article, a special Fourier mode version of the standard path integral was developed. Here we illustrate how to use this implementation by obtaining the exact Boltzmann operator matrix elements of the harmonic oscillator.

We have derived the following general expression for the off-diagonal trace of the Boltzmann operator:

$$\Delta = \int_{-\infty}^{\infty} dx \int_{-\infty}^{\infty} dy < x|\exp(-\beta\hat{H})|y >$$

$$= \int da_0 \prod_{j=1}^{\infty} \int da_j (\frac{\omega_j^2 \beta M}{4\pi})^{\frac{1}{2}} \exp(-\frac{M}{4}\beta\Sigma_{j=0}^{\infty}\omega_j^2 a_j^2 - \int_0^{\beta\hbar} d\tau V(\Sigma_{j=0}^{\infty}\cos(\omega_j\tau)a_j)/\hbar), \tag{1}$$

with $\omega_j = \frac{j\pi}{\beta\hbar}$. The path is

$$x(\tau) = \Sigma_{j=0}^{\infty}\cos(\omega_j\tau)a_j,\ 0 \le \tau \le \beta\hbar. \tag{2}$$

This path integral includes all open paths, ending and starting at arbitrary places.

If we want to calculate just $< x|\exp(-\beta\hat{H})|y >$ rather than Δ we may add delta functions to Eq. 1: $\delta(y - x(0)) = \int d\theta_1 \exp(i\theta_1(y - x(0)))/2\pi = \int d\theta_1 \exp(i\theta_1(y - a_0 - a_1 - ..))/2\pi$ and $\delta(x - x(\beta\hbar)) = \int d\theta_2 \exp(i\theta_2(x - a_0 + a_1 - a_2 + ..))/2\pi$. Doing this and considering a harmonic oscillator we get

$$< x|\exp(-\beta\hat{H})|y >$$

$$= \int d\theta_1 \int d\theta_2 \int da_0 \prod_{j=1}^{\infty} \int da_j (\frac{\omega_j^2 \beta M}{4\pi})^{\frac{1}{2}} \exp(i\theta_1(y - a_0 - a_1 - ..))/2\pi$$

$$\times \exp(i\theta_2(x - a_0 + a_1 - a_2 + ..))/2\pi$$

$$\times \exp(-\frac{M}{4}\beta\Sigma_{j=0}^{\infty}\omega_j^2 a_j^2 - \int_0^{\beta\hbar} d\tau \frac{1}{2}M\Omega^2(\Sigma_{j=0}^{\infty}\cos(\omega_j\tau)a_j)^2/\hbar)$$

$$= (\frac{1}{2\pi})^2 \int d\theta_1 \int d\theta_2 \int da_0 \prod_{j=1}^{\infty} \int da_j (\frac{\omega_j^2\beta M}{4\pi})^{\frac{1}{2}} \exp(i\theta_1(y - a_0 - a_1 - ..)) \quad (3)$$

$$\times \exp(i\theta_2(x - a_0 + a_1 - a_2 + ..)) \times \exp(-\frac{M}{4}\beta\Sigma_{j=0}^{\infty}(\omega_j^2 + \epsilon_{0j}\Omega^2)a_j^2),$$

where $\epsilon_{0j} = 1$ for $j \neq 0$ and $\epsilon_{0j} = 2$ for $j = 0$. Next we perform a completion of squares and integrate out all Fourier modes

$$< x|\exp(-\beta\hat{H})|y >$$

$$= (\frac{1}{2\pi})^2 \int d\theta_1 \int d\theta_2 \int da_0 \prod_{j=1}^{\infty} \int da_j (\frac{\omega_j^2\beta M}{4\pi})^{\frac{1}{2}} \exp(i\theta_1 y + i\theta_2 x)$$

$$\times \exp(-\frac{M}{4}\beta\Sigma_{j=0,2,4,..}^{\infty}(\omega_j^2+\epsilon_{0j}\Omega^2)(a_j+\frac{2i(\theta_2+\theta_1)}{M\beta(\omega_j^2+\epsilon_{0j}\Omega^2)})^2)\times\exp(-\frac{1}{M\beta}\Sigma_{j=0,2,4,..}^{\infty}\frac{(\theta_2+\theta_1)^2}{\omega_j^2+\epsilon_{0j}\Omega^2}) \quad (4)$$

$$\times \exp(-\frac{M}{4}\beta\Sigma_{j=1,3,5,..}^{\infty}(\omega_j^2+\Omega^2)(a_j+\frac{2i(\theta_1-\theta_2)}{M\beta(\omega_j^2+\Omega^2)})^2)\times\exp(-\frac{1}{M\beta}\Sigma_{j=1,3,5,..}^{\infty}\frac{(\theta_1-\theta_2)^2}{\omega_j^2+\Omega^2}),$$

or

$$< x|\exp(-\beta\hat{H})|y >$$

$$= (\frac{1}{2\pi})^2(\frac{2\pi}{M\beta\Omega^2})^{1/2}\prod_{j=1}^{\infty}(\frac{\omega_j^2}{\omega_j^2+\Omega^2})^{1/2}$$

$$\times \int d\theta_1 \int d\theta_2 \exp(i\theta_1 y + i\theta_2 x) \times \exp(-\frac{1}{M\beta}\Sigma_{j=0,2,4,..}^{\infty}\frac{(\theta_2+\theta_1)^2}{\omega_j^2+\epsilon_{0j}\Omega^2})$$

$$\times \exp(-\frac{1}{M\beta}\Sigma_{j=1,3,5,..}^{\infty}\frac{(\theta_1-\theta_2)^2}{\omega_j^2+\Omega^2}). \quad (5)$$

Thereafter we use an identity which is valid for the so-called Matsubara frequencies[1] $\tilde{\omega}_j = 2\pi j/\beta\hbar$:

$$\prod_{j=1}^{\infty}\frac{\tilde{\omega}_j^2}{\tilde{\omega}_j^2+\Omega^2} = \frac{\beta\hbar\Omega/2}{\sinh(\beta\hbar\Omega/2)}. \quad (6)$$

Notice that these frequencies are twice as large as our ω_j's. We may therefore write:

$$\prod_{j=1}^{\infty}\frac{4\omega_j^2}{4\omega_j^2+\Omega^2} = \frac{\beta\hbar\Omega/2}{\sinh(\beta\hbar\Omega/2)} \Leftrightarrow \prod_{j=1}^{\infty}\frac{\omega_j^2}{\omega_j^2+(\Omega/2)^2} = \frac{\beta\hbar\Omega/2}{\sinh(\beta\hbar\Omega/2)}. \quad (7)$$

Thus, we obtain:

$$(\frac{2\pi}{M\beta\Omega^2})^{1/2}\prod_{j=1}^{\infty}(\frac{\omega_j^2}{\omega_j^2+\Omega^2})^{1/2}=(\frac{2\pi}{M\beta\Omega^2}\frac{\beta\hbar\Omega}{\sinh(\beta\hbar\Omega)})^{1/2}. \tag{8}$$

Then we get

$$\begin{aligned}&< x|\exp(-\beta\hat{H})|y>\\ &=(\frac{1}{2\pi})^2(\frac{2\pi}{M\beta\Omega^2}\frac{\beta\hbar\Omega}{\sinh(\beta\hbar\Omega)})^{1/2}\\ &\times\int d\theta_1\int d\theta_2\exp(i\theta_1 y+i\theta_2 x)\times\exp(-\frac{1}{M\beta}\Sigma_{j=0,2,4,..}^{\infty}\frac{(\theta_2+\theta_1)^2}{\omega_j^2+\epsilon_{0j}\Omega^2})\\ &\times\exp(-\frac{1}{M\beta}\Sigma_{j=1,3,5,..}^{\infty}\frac{(\theta_1-\theta_2)^2}{\omega_j^2+\Omega^2}).\end{aligned} \tag{9}$$

Next we change integration variables to $\lambda=(\theta_1+\theta_2)/2$ and $\Delta=\theta_2-\theta_1$. We now obtain

$$\begin{aligned}&< x|\exp(-\beta\hat{H})|y>\\ &=(\frac{1}{2\pi})^2(\frac{2\pi}{M\beta\Omega^2}\frac{\beta\hbar\Omega}{\sinh(\beta\hbar\Omega)})^{1/2}\\ &\times\int d\lambda\int d\Delta\exp(i(\lambda-\Delta/2)y+i(\lambda+\Delta/2)x)\times\exp(-\frac{4}{M\beta}\Sigma_{j=0,2,4,..}^{\infty}\frac{\lambda^2}{\omega_j^2+\epsilon_{0j}\Omega^2})\\ &\times\exp(-\frac{1}{M\beta}\Sigma_{j=1,3,5,..}^{\infty}\frac{\Delta^2}{\omega_j^2+\Omega^2}).\end{aligned} \tag{10}$$

To proceed, we consider the Feynman-Kleinert smearing width for a harmonic oscillator with frequency Ω at inverse temperature $\beta=1/k_BT$. It is given by[1]

$$a_{FK}^2(\Omega,\beta)=2\frac{k_BT}{M}\Sigma_{j=1}^{\infty}\frac{1}{\tilde{\omega}_j^2+\Omega^2}, \tag{11}$$

where the Matsubara frequencies $\tilde{\omega}_j=2\pi j/\beta\hbar$ have appeared again. We then see that

$$\begin{aligned}&\frac{4}{M\beta}\Sigma_{j=0,2,4,..}^{\infty}\frac{1}{\omega_j^2+\Omega^2}=\frac{4}{M\beta}\Sigma_{j=2,4,..}^{\infty}\frac{1}{\omega_j^2+\Omega^2}+\frac{4}{M\beta\Omega^2}\\ &=\frac{4}{M\beta}\Sigma_{j=1}^{\infty}\frac{1}{\tilde{\omega}_j^2+\Omega^2}+\frac{4}{M\beta\Omega^2}=2a_{FK}^2(\Omega,\beta)+\frac{4}{M\beta\Omega^2}.\end{aligned} \tag{12}$$

A similar rewriting shows that

$$\frac{4}{M\beta}\Sigma_{j=0,2,4,..}^{\infty}\frac{\lambda^2}{(\omega_j^2+\epsilon_{0j}\Omega^2)}=2\lambda^2a_{FK}^2(\Omega,\beta)+\frac{2\lambda^2}{M\beta\Omega^2}. \tag{13}$$

$a^2_{FK}(\Omega,\beta)$ can also be written as[1]:

$$a^2_{FK}(\Omega,\beta) = \frac{k_BT}{M\Omega^2}[\frac{\hbar\Omega}{2k_BT}\coth(\frac{\hbar\Omega}{2k_BT}) - 1]. \quad (14)$$

Substituting Eq. 14 into Eq. 13 leads to

$$\frac{4}{M\beta}\Sigma^{\infty}_{j=0,2,4,..}\frac{\lambda^2}{(\omega_j^2 + \epsilon_{0j}\Omega^2)}(\Omega,\beta) = \frac{\lambda^2\hbar}{M\Omega}\coth(\frac{\hbar\Omega}{2k_BT}) \equiv A\lambda^2. \quad (15)$$

Let us now set $B \equiv \frac{1}{M\beta}\Sigma^{\infty}_{j=1,3,5,..}\frac{1}{(\omega_j^2+\Omega^2)}$ and then we simplify this expression. We begin by writing:

$$\begin{aligned} M\beta \times B = \Sigma^{\infty}_{j=1,3,5,..}\frac{1}{(\omega_j^2+\Omega^2)} &= \Sigma^{\infty}_{j=1}\frac{1}{(\omega_j^2+\Omega^2)} - \Sigma^{\infty}_{j=2,4,..}\frac{1}{(\omega_j^2+\Omega^2)} \\ &= 4\Sigma^{\infty}_{j=1}\frac{1}{((2\omega_j)^2+(2\Omega)^2)} - \Sigma^{\infty}_{j=2,4,..}\frac{1}{(\omega_j^2+\Omega^2)} \quad (16) \\ &= 4\Sigma^{\infty}_{j=2,4,..}\frac{1}{({\omega_j}^2+(2\Omega)^2)} - \Sigma^{\infty}_{j=2,4,..}\frac{1}{(\omega_j^2+\Omega^2)}. \end{aligned}$$

Next, we use Eq. 12 twice. First to get:

$$\frac{4}{M\beta}\Sigma^{\infty}_{j=2,4,..}\frac{1}{(\omega_j^2+(2\Omega)^2)} = 2a^2_{FK}(2\Omega,\beta) \quad (17)$$

and then again to also get

$$\frac{1}{M\beta}\Sigma^{\infty}_{j=2,4,..}\frac{1}{(\omega_j^2+\Omega^2)} = \frac{1}{2}a^2_{FK}(\Omega,\beta).$$

Now we can write:

$$\begin{aligned} B &= 2a^2_{FK}(2\Omega,\beta) - \frac{1}{2}a^2_{FK}(\Omega,\beta) \\ &= \frac{1}{2}\frac{k_BT}{M\Omega^2}[\frac{\hbar\Omega}{k_BT}\coth(\frac{\hbar\Omega}{k_BT}) - 1] - \frac{1}{2}\frac{k_BT}{M\Omega^2}[\frac{\hbar\Omega}{2k_BT}\coth(\frac{\hbar\Omega}{2k_BT}) - 1] \\ &= \frac{\hbar}{2M\Omega}\{\coth(\frac{\hbar\Omega}{k_BT}) - \frac{1}{2}\coth(\frac{\hbar\Omega}{2k_BT})\}. \quad (18) \end{aligned}$$

Thereafter we use the identity

$$\coth(2x) - \frac{1}{2}\coth(x) = \frac{1}{2}\tanh(x), \quad (19)$$

to obtain

$$B = \frac{\hbar}{M\Omega}\frac{1}{4}\tanh(\frac{\hbar\Omega}{2k_BT}). \quad (20)$$

Finally we return to $< x|\exp(-\beta\hat{H})|y >$, where we will integrate over λ and Δ. We write:

$$< x|\exp(-\beta\hat{H})|y >$$

$$= (\frac{1}{2\pi})^2 (\frac{2\pi}{M\beta\Omega^2} \frac{\beta\hbar\Omega}{\sinh(\beta\hbar\Omega)})^{1/2}$$

$$\times \int d\lambda \int d\Delta \exp(-A(\lambda - \frac{i(x+y)/2}{A})^2) \times \exp(-\frac{(x+y)^2}{4A})$$

$$\times \exp(-B(\Delta - \frac{i(x-y)/4}{B})^2) \times \exp(-\frac{(x-y)^2}{16B})$$

$$= (\frac{1}{2\pi})^2 (\frac{2\pi}{M\beta\Omega^2} \frac{\beta\hbar\Omega}{\sinh(\beta\hbar\Omega)})^{1/2} (\frac{\pi^2}{AB})^{1/2} \quad (21)$$

$$\times \exp(-\frac{(x+y)^2}{4A}) \times \exp(-\frac{(x-y)^2}{16B}).$$

Next we replace A and B using Eqs. 15 and 20 to get

$$< x|\exp(-\beta\hat{H})|y >$$

$$= (\frac{1}{2\pi})^2 (\frac{2\pi}{M\beta\Omega^2} \frac{\beta\hbar\Omega}{\sinh(\beta\hbar\Omega)})^{1/2} (\frac{\pi^2}{\frac{\hbar}{M\Omega}\coth(\frac{\hbar\Omega}{2k_BT})\frac{\hbar}{M\Omega}\frac{1}{4}\tanh(\frac{\hbar\Omega}{2k_BT})})^{1/2}$$

$$\times \exp(-\frac{(x+y)^2}{\frac{4\hbar}{M\Omega}\coth(\frac{\hbar\Omega}{2k_BT})}) \times \exp(-\frac{(x-y)^2}{\frac{4\hbar}{M\Omega}\tanh(\frac{\hbar\Omega}{2k_BT})}) \quad (22)$$

$$= (\frac{M\Omega}{\pi 2\hbar \sinh(\beta\hbar\Omega)})^{1/2} \times \exp(-\frac{(x+y)^2}{\frac{4\hbar}{M\Omega}\coth(\frac{\hbar\Omega}{2k_BT})}) \times \exp(-\frac{(x-y)^2}{\frac{4\hbar}{M\Omega}\tanh(\frac{\hbar\Omega}{2k_BT})}).$$

Using $\cosh^2(x) - \sinh^2(x) = 1$, $\cosh^2(x) + \sinh^2(x) = \cosh(2x)$ and $2\sinh(x)\cosh(x) = \sinh(2x)$, we may finally rewrite Eq. 22 into

$$< x|\exp(-\beta\hat{H})|y >$$

$$= (\frac{M\Omega}{\pi 2\hbar \sinh(\beta\hbar\Omega)})^{1/2} \times \exp(-\frac{M\Omega}{2\hbar\sinh(\frac{\hbar\Omega}{k_BT})}[(x^2+y^2)\cosh(\frac{\hbar\Omega}{k_BT}) - 2xy]), \quad (23)$$

which is the exact expression, see e.g. [2].

References

[1] Kleinert; H., in *Path Integrals in Quantum Mechanics, Statistics, Polymer Physics, and Financial Markets*, fifth edition, World Scientific, Singapore, 2009.

[2] Feynman; R. P., in *Statistical Mechanics*: *A set of Lectures*, Addison-Wesley, Massachusetts, 1998.
